# Outcomes of mild-to-moderate postresuscitation shock after non-shockable cardiac arrest and association with temperature management: a post hoc analysis of HYPERION trial data

**DOI:** 10.1186/s13613-022-01071-z

**Published:** 2022-10-17

**Authors:** Ines Ziriat, Aurélie Le Thuaut, Gwenhael Colin, Hamid Merdji, Guillaume Grillet, Patrick Girardie, Bertrand Souweine, Pierre-François Dequin, Thierry Boulain, Jean-Pierre Frat, Pierre Asfar, Bruno Francois, Mickael Landais, Gaëtan Plantefeve, Jean-Pierre Quenot, Jean-Charles Chakarian, Michel Sirodot, Stéphane Legriel, Nicolas Massart, Didier Thevenin, Arnaud Desachy, Arnaud Delahaye, Vlad Botoc, Sylvie Vimeux, Frederic Martino, Jean Reignier, Alain Cariou, Jean Baptiste Lascarrou

**Affiliations:** 1 Médecine Intensive Réanimation, University Hospital Centre, Nantes, France; 2 Direction de la Recherche Clinique et l’Innovation, Plateforme de Méthodologie et Biostatistique, University Hospital Centre, Nantes, France; 3 Medecine Intensive Reanimation, District Hospital Center, La Roche-sur-Yon, France; 4 AfterROSC Network, Paris, France; 5 Université de Strasbourg (UNISTRA), Faculté de Médecine; Hôpitaux Universitaires de Strasbourg, Nouvel Hôpital Civil, Service de Médecine Intensive Réanimation, Strasbourg, France; 6 INSERM (French National Institute of Health and Medical Research), UMR 1260, Regenerative Nanomedicine (RNM), FMTS, Strasbourg, France; 7 Medical Intensive Care Unit, South Brittany General Hospital Centre, Lorient, France; 8 Médecine Intensive Réanimation, CHU Lille, 59000 Lille, France; 9 Faculté de Médicine, Université de Lille, 59000 Lille, France; 10 Medical Intensive Care Unit, University Hospital Centre, Clermond-Ferrand, France; 11 INSERM CIC1415, CHRU de Tours, Tours, France; 12 Medical Intensive Care Unit, University Hospital Centre, Tours, France; 13 Inserm UMR 1100 – Centre d’Étude des Pathologies Respiratoires, Tours University, Tours, France; 14 Medical Intensive Care Unit, Regional Hospital Centre, Orleans, France; 15 Médecine Intensive Réanimation, CHU de Poitiers, Poitiers, France; 16 INSERM, CIC-1402, ALIVES, Poitiers, France; 17 Université de Poitiers, Faculté de Médecine et de Pharmacie de Poitiers, Poitiers, France; 18 Medical Intensive Care Unit, University Hospital Centre, Angers, France; 19 Service de Réanimation Polyvalente, University Hospital Centre, Limoges, France; 20 INSERM CIC 1435 & UMR 1092, University Hospital Centre, Limoges, France; 21 Medical-Surgical Intensive Care Unit, Community Hospital Centre, Le Mans, France; 22 Medical-Surgical Intensive Care Unit, Community Hospital Centre, Argenteuil, France; 23 Medical Intensive Care Unit, University Hospital Centre, Dijon, France; 24 Medical-Surgical Intensive Care Unit, Community Hospital Centre, Roanne, France; 25 Medical-Surgical Intensive Care Unit, Community Hospital Centre, Annecy, France; 26 Medical-Surgical Intensive Care Unit, Versailles Hospital, Versailles, France; 27 Medical-Surgical Intensive Care Unit, Community Hospital Centre, Saint Brieuc, France; 28 Medical-Surgical Intensive Care Unit, Community Hospital Centre, Lens, France; 29 Medical-Surgical Intensive Care Unit, Community Hospital Centre, Angoulême, France; 30 Medical-Surgical Intensive Care Unit, Community Hospital Centre, Rodez, France; 31 Medical-Surgical Intensive Care Unit, Community Hospital Centre, Saint Malo, France; 32 Medical-Surgical Intensive Care Unit, Community Hospital Centre, Montauban, France; 33 Medical Intensive Care Unit, University Hospital Centre, Pointe-à-Pitre, France; 34 Medical Intensive Care Unit, Cochin Hospital (APHP) and University of Paris, Paris, France; 35 Paris Cardiovascular Research Centre, INSERM U970, Paris, France; 36 Service de Médecine Intensive Réanimation, Centre Hospitalier Universitaire, 30 Boulevard Jean Monnet, 44093 Nantes Cedex 1, France

**Keywords:** Cardiac arrest, Targeted temperature management, Therapeutic hypothermia, In-hospital, Postresuscitation shock

## Abstract

**Background:**

Outcomes of postresuscitation shock after cardiac arrest can be affected by targeted temperature management (TTM). A post hoc analysis of the “TTM1 trial” suggested higher mortality with hypothermia at 33 °C. We performed a post hoc analysis of HYPERION trial data to assess potential associations linking postresuscitation shock after non-shockable cardiac arrest to hypothermia at 33 °C on favourable functional outcome.

**Methods:**

We divided the patients into groups with vs. without postresuscitation (defined as the need for vasoactive drugs) shock then assessed the proportion of patients with a favourable functional outcome (day-90 Cerebral Performance Category [CPC] 1 or 2) after hypothermia (33 °C) vs. controlled normothermia (37 °C) in each group. Patients with norepinephrine or epinephrine > 1 µg/kg/min were not included.

**Results:**

Of the 581 patients included in 25 ICUs in France and who did not withdraw consent, 339 had a postresuscitation shock and 242 did not. In the postresuscitation-shock group, 159 received hypothermia, including 14 with a day-90 CPC of 1–2, and 180 normothermia, including 10 with a day-90 CPC of 1–2 (8.81% vs. 5.56%, respectively; *P* = 0.24). After adjustment, the proportion of patients with CPC 1–2 also did not differ significantly between the hypothermia and normothermia groups (adjusted hazards ratio, 1.99; 95% confidence interval, 0.72–5.50; *P* = 0.18). Day-90 mortality was comparable in these two groups (83% vs. 86%, respectively; *P* = 0.43).

**Conclusions:**

After non-shockable cardiac arrest, mild-to-moderate postresuscitation shock at intensive-care-unit admission did not seem associated with day-90 functional outcome or survival. Therapeutic hypothermia at 33 °C was not associated with worse outcomes compared to controlled normothermia in patients with postresuscitation shock.

*Trial registration* ClinicalTrials.gov, NCT01994772

**Supplementary Information:**

The online version contains supplementary material available at 10.1186/s13613-022-01071-z.

## Introduction

Postcardiac arrest syndrome is a complex combination of pathophysiological processes, including brain injury, myocardial dysfunction, and systemic ischemia/reperfusion injury with vasoplegia. A major component of this syndrome is hemodynamic instability, which compromises the perfusion of vital organs. Postresuscitation shock (PRS) develops in 15% to 50% of patients admitted to the intensive care unit (ICU) after the return of spontaneous circulation (ROSC) [1, 2] and accounts for nearly a third of in-ICU deaths [3]. PRS is also associated with worse functional outcomes [4, 5].

Information on targeted temperature management (TTM) of patients with PRS at ICU admission is discordant. In vitro and animal studies have documented favourable inotropic effects of hypothermia on the myocardium [6, 7], suggesting possible benefits on haemodynamics. In patients with cardiogenic shock, hypothermia may improve cardiac function, thereby increasing the cardiac index, arterial blood pressure and, possibly, central venous oxygen saturation (ScvO_2_), while decreasing the lactate level and vasopressor requirements [8–11]. This global hemodynamic improvement might improve survival and functional outcomes after cardiac arrest by restoring adequate global and cerebral perfusion [12]. However, recent work suggests opposite effects. In a post hoc study of the “TTM1 trial”, compared to TTM at 36 °C, hypothermia at 33 °C was associated with a lower heart rate, higher lactate level, and greater vasopressor requirements [13]. In another post hoc analysis of the same trial, in the patient sub-group with PRS at ICU admission, hypothermia at 33 °C was not associated with better survival or less severe PRS severity, and there was a trend toward worse outcomes in the 33 °C group, significant after multivariable adjustments [14]. However, most of those studies were dedicated to patient with cardiac arrest from cardiac cause, whereas cardiac arrest from non-cardiac cause is increasingly frequently and potentially associated with different physiopathology [15]. Last patient sub-group with PRS was generally excluded from initial randomized trials of therapeutic hypothermia after cardiac arrest [16, 17], resulting in a relative lack of safety and efficacy data. Current guidelines do not include clear recommendations for patients with PRS [18].

The objective of this post hoc analysis of data from the HYPERION randomized trial comparing 33° to controlled normothermia (37 °C) after non-shockable cardiac arrest was to determine whether the intervention was associated with the day-90 functional outcome specifically in the sub-group of patients with PRS.

## Methods

### Patients

HYPERION was a randomized, multicenter, controlled trial with blinded outcome assessment that included adults (≥ 18 years) who achieved the ROSC after out-of-hospital or in-hospital cardiac arrest in a non-shockable rhythm, due to any cause, and who were comatose (Glasgow Coma Scale score ≤ 8) at ICU admission (NCT01994772) [2, 19]. The main exclusion criteria were a no-flow time longer than 10 min; a low-flow time longer than 60 min, a time from cardiac arrest to screening longer than 300 min; and major hemodynamic instability defined as a continuous epinephrine or norepinephrine infusion > 1 µg/kg/min.

### Targeted temperature management (TTM)

Patients were randomized to moderate therapeutic hypothermia at 33 °C or controlled normothermia (37 °C). In the hypothermia group, cooling to 33 °C was induced then maintained for 24 h, after which slow rewarming at a rate no faster than 0.5 °C/h was provided to a body temperature of 36.5–37.5 °C, which was maintained for 24 h. In the normothermia group, body temperature was maintained at 36.5–37.5 °C for 48 h. Sedation was tapered when the body temperature rose above 36 °C in the hypothermia group and after 12 h in the normothermia group.

### Definition of postresuscitation shock (PRS)

PRS was defined as a systolic blood pressure below 90 mm Hg for at least 30 min with impaired end-organ perfusion (cool extremities, mottling, or urine output < 30 mL/h), requiring norepinephrine and/or epinephrine intravenous infusion. PRS was recorded at ICU admission. Severe shock requiring a continuous epinephrine or norepinephrine infusion > 1 µg/kg/min was an non-inclusion criterion in the HYPERION trial.

### Hemodynamic management

Hemodynamic evaluations were conducted to allow blood volume optimization. Hypovolemia was managed with crystalloid or colloid infusion, according to standard practice in each participating ICU. Vasoactive drug treatment was at the discretion of the physicians, who followed international guidelines and local protocols. The targets were 65 mmHg for mean arterial pressure (MAP) and, if measured, ≥ 70% for ScvO_2_.

### Outcomes

The primary outcome was a favourable day-90 functional outcome defined as a Cerebral Performance Category (CPC) score of 1 of 2. CPC scores can range from 1 to 5, with higher scores indicating greater disability. A score of 1 indicates good cerebral performance or minor disability and a score of 2 moderate disability.

Secondary outcomes were day-90 mortality and PRS severity assessed based on the heart rate, MAP, cardiovascular SOFA-score component, and serum lactate level.

### Statistics

Categorical data were described as frequency (percentage) and compared using the Chi-square test or Fisher’s exact test, as appropriate. Continuous data were described as median [interquartile range] and compared by applying Student’s *t* test if normally distributed and the Wilcoxon signed-rank test otherwise.

The primary outcome (CPC 1 or 2) was compared between groups with vs. without PRS using logistic regression. The Kaplan–Meier method was applied to assess 90-day survival and Cox regression to identify predictors of day-90 mortality by computing hazard ratios (HRs) with their 95% confidence intervals (95% CIs) with adjustment on CAHP score. CAHP score is a score dedicated to early evaluation of brain damage with prediction of poor functional outcome at hospital discharge [20]. It has been validated both for out-of-hospital cardiac arrest but also in-hospital cardiac arrest [21]. Changes over time in heart rate, MAP, the SOFA score, and lactate level were assessed by generalized linear mixed-effects models.

No imputation strategy was used for missing data. *P* values < 0.05 were considered significant.

All analyses were performed using SAS 9.4 (SAS Institute, Cary, NC).

## Results

On the 584 patients included in the HYPERION trial, 3 withdrew (all in the hypothermia group) leaving 581 patients for analysis: 339 (58%) had PRS at ICU admission, 159 in the hypothermia group and 180 in the normothermia group (Additional file 1: Fig. S1).

### Characteristics and outcomes in the groups with (*n* = 339) vs. without (*n* = 242) shock

Compared to the group without PRS at ICU admission, the group with PRS had a lower Glasgow Coma Scale score, higher proportion of patients given bystander resuscitation, longer low-flow time, and higher serum lactate at ICU admission (Additional file 1: Table S1). Mortality by day 90 was 84.66%, compared to 78.93% in the group without PRS (*P* = 0.07). The proportions of patients with day-90 CPC 1–2 did not differ significantly according to presence or absence of PRS (7.08% vs. 9.09%, respectively; *P* = 0.37).

### Outcomes with hypothermia (*n* = 159) vs. normothermia (*n* = 180) in the sub-group with postresuscitation shock (PRS)

**Fig. 1:**
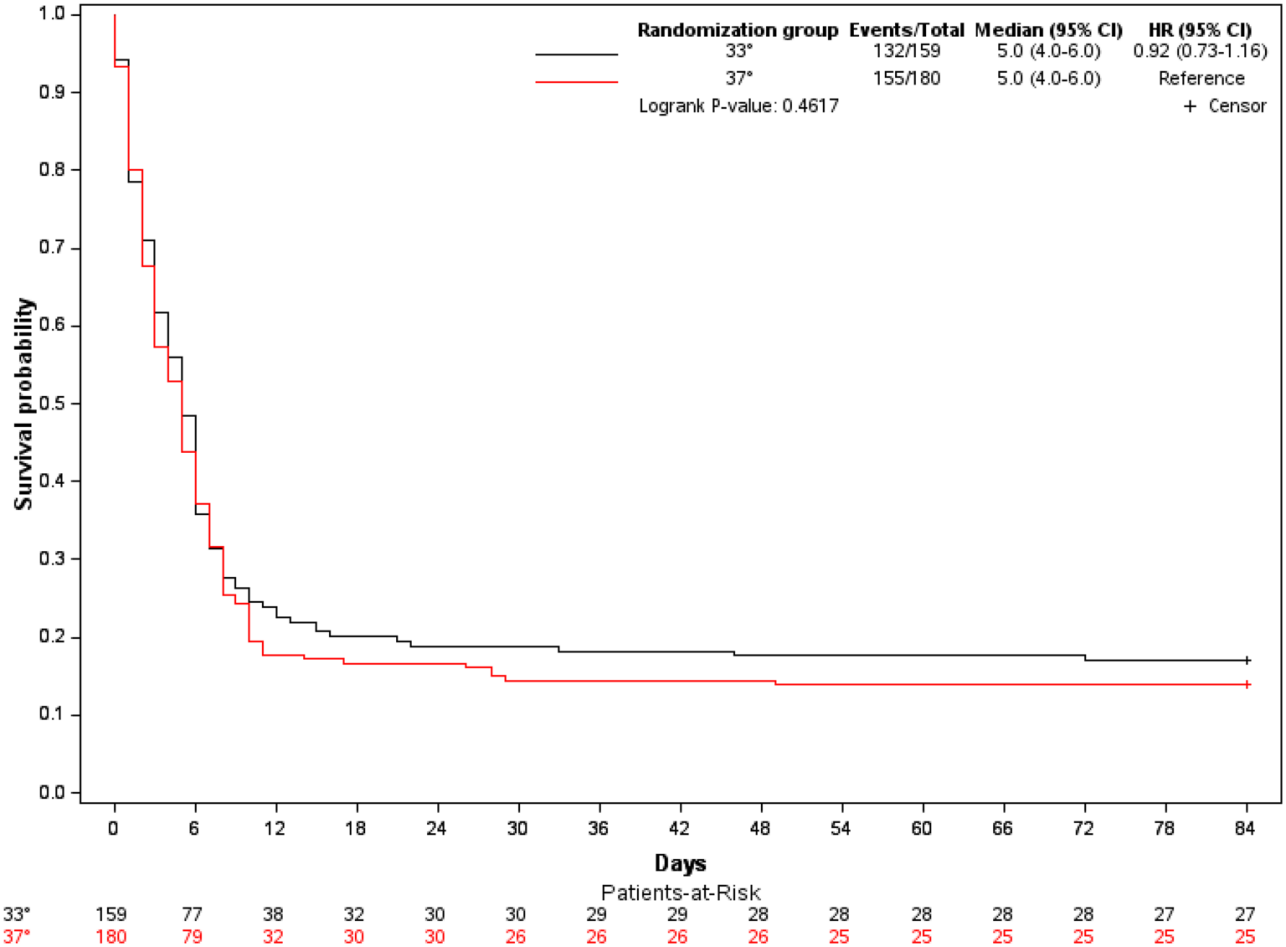
Probability of survival to 90 days according to temperature allocated in the subgroup of patients with PRS. HR: hazard ratio; 95% CI: 95% confidence interval. PRS: postresuscitation shock

**Fig. 2:**
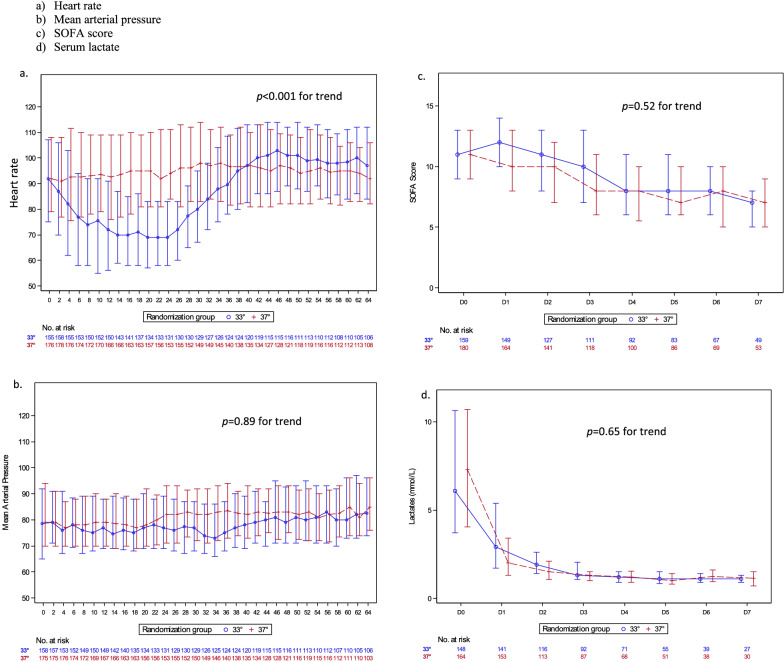
Changes over time in physiological variables according to target temperature in the patients with postresuscitation shock (PRS) at admission. SOFA: Sequential Organ Failure Assessment. The lactate level was determined at admission to the intensive care unit

On day 90, the number of patients with a CPC score of 1 or 2 on day 90 was 14/159 (8.81%) in the hypothermia group and 10/180 (5.56%) in the normothermia group (*P* = 0.24) (Table 1). After adjustment on the Cardiac Arrest Hospital Prognosis (CAHP) score, the proportion of CPC 1–2 patients on day 90 was also not significantly different between the two TTM groups (Additional file 1: Table S2). Neither was day-90 mortality different (83% with hypothermia and 86% with normothermia, *P* = 0.43, Fig. 1 and Table 2). Heart rate was significantly lower in the hypothermia group but no significant differences existed for MAP, the SOFA score, or the serum lactate level (Fig. 2).

**Table 1:** Baseline characteristics of patients with postresuscitation shock (PRS) managed with hypothermia (33 °C) or normothermia (37 °C)

Characteristics	33° (*N* = 159)	37° (*N* = 180)	*P* value
Age, years, median [IQR)	67.1 [56.9–74.9]	67.8 [59.5–77.1]	0.23
Males, *n* (%)	99 (62.2)	111 (61.7)	0.91
Charlson score, median [IQR)	4 (2–6)	4 (2–6)	0.37
Comorbidities, *n* (%)			
Cardiovascular	94/118 (79.7)	116/139 (83.5)	0.43
Pulmonary	56/118 (47.4)	66/139 (47.5)	0.10
Cardiac arrest location, *n* (%)			0.001
Home	74/159 (46.5)	95/180 (52.8)	
Public place	52/159 (32.7)	29/180 (16.1)	
Hospital	33/159 (20.8)	56/180 (31.1)	
Arrest witnessed, *n* (%)	154/159 (96.9)	170/179 (95.0)	0.39
Basic life support provided by bystander, *n* (%)	120/153 (78.4)	133/171 (77.8)	0.89
First-recorded cardiac rhythm, *n* (%)			0.97
Asystole	125/143 (87.4)	148/169 (87.6)	
Pulseless electrical activity	18/143 (12.6)	21/169 (12.4)	
Cause of cardiac arrest, *n* (%)			*
Cardiac	43/159 (27.0)	50/180 (27.8)	
Asphyxia	81/159 (51.0)	9/180 (50.5)	
Anaphylaxis	4/159 (2.5)	3/180 (1.7)	
Neurological	5/159 (3.1)	4/180 (2.2)	
Other medical cause	15/159 (9.4)	16/180 (8.9)	
Pulmonary embolism	8/159 (5.0)	9/180 (5.0)	
Trauma	1/159 (0.6)	2/180 (1.1)	
Drug poisoning	0/159 (0.0)	4/180 (2.2)	
Drowning	2/159 (1.3)	1/180 (0.6)	
GCS score, median [IQR]	3 [3–3	3 [3–3]	0.62
Time to randomisation, min, median [IQR]	241.0 [192.0–285.0]	226.0 [178.5–276.5]	0.21
Temperature, °C, median [IQR]	35.5 [34.3–36.3]	35.4 [34.4–36.5]	0.86
No-flow time, min, median [IQR]	2 [0–5]	0 [0–5]	0.10
Low-flow time, min, median [IQR]	19 [10–28]	19.0 [10–28]	0.82
Serum lactate at admission, mmol/L, median [IQR]	6.1 [3.7–10.6]	7.3 [4.0–10.7]	0.40
Epinephrine injection, *n* (%)	150/159 (94.3)	174/180 (96.7)	0.30
Total epinephrine dose, mg, median [IQR]	3 (2, 5)	4 (2, 5)	0.44
PCI, *n* (%)	13/159 (8.2)	13/180 (7.2)	0.74
CAHP score, median [IQR]	190 [170–212]	195 [177–222]	0.073
Type of vasoactive drug at admission, *n* (%)			
Norepinephrine	90/159 (56.6)	93/180 (51.7)	0.36
Epinephrine	83/159 (52.2)	100/180 (55.6)	0.54
Dobutamine	7/159 (4.4)	11/180 (6.1)	0.48
Dopamine	0/159 (0)	1/180 (0.6)	1.00
Early onset pneumonia, *n* (%)	35 (22.0)	40 (22.2)	0.96

GCS: Glasgow Coma Scale; CAHP: Cardiac Arrest Hospital Prognosis score; PCI: percutaneous coronary intervention

*Too many categories for a meaningful statistical analysis

**Table 2:** Outcomes of patients with postresuscitation shock managed with hypothermia (33 °C) vs. normothermia (37 °C)

Characteristics	33° (*N* = 159)	37° (*N* = 180)	*P* value
CPC, *n* (%)			0.45
1	8 (5.0)	8 (4.5)	
2	6 (3.8)	2 (1.1)	
3	13 (8.2)	15 (8.3)	
4	0 (0.0)	0 (0.0)	
5	132 (83.0)	155 (86.1)	
Favourable outcome (CPC 1 or 2) on day 90, *n* (%)	14 (8.8)	10 (5.6)	0.24
90-day mortality, *n* (%)	132 (83.0)	155 (86.1)	0.43

CPC: Cerebral Performance Category

### Factors associated with the day-90 functional outcome

The group with unfavourable day-90 functional outcomes (CPC 3, 4, or 5; *n* = 315) had a larger proportion of patients with cardiac arrest at home, longer no-flow and low-flow times, higher epinephrine doses, and a higher CAHP score (Table 3).

**Table 3:** Characteristics of patients with postresuscitation shock (PRS) in the groups with favourable vs. unfavourable outcome^a^

Characteristics	Favourable^a^ outcome (*N* = 24)	Unfavourable^a^ outcome (*N* = 315)	*P* value
Age, years, median [IQR]	62.5 [57.1–69.8]	67.6 [58.0–77.2]	0.085
Males, *n* (%)	19 (79.2)	191 (60.6)	0.072
Cardiac arrest location, *n* (%)			0.012
Home	6 (25.0)	163 (51.7)	
Elsewhere	18 (75.0)	152 (48.3)	
No-flow time, min, median [IQR]	0.0 [0.0–2.0]	2.0 [0.0–5.0]	0.012
Low-flow time, min, median [IQR]	10.0 [5.0–12.5]	20.0 [12.0–29.0]	< 0.0001
Arterial pH, median [IQR]	7.2 [7.0–7.3]	7.2 [7.1–7.3]	0.79
PCI, *n* (%)	1 (4.2)	25(7.9)	1.00
Total epinephrine dose, mg, *n* (%)			0.021
0	4 (17.4)	11 (3.5)	
1 or 2	8 (34.8)	104 (33.3)	
≥ 3	11 (47.8)	197 (63.1)	
CAHP score, median [IQR]	171 [150–185]	196 [175–219]	0.0001
33° group^b^, *n* (%)	14 (58.3)	145 (46.0)	0.24
GCS score, median [IQR]	3 [3–3]	3 [3–3]	0.58

PCI: percutaneous coronary intervention; CAHP: Cardiac Arrest Hospital Prognosis; GCS: Glasgow Coma Scale

^a^Favourable outcome was defined as a Cerebral Performance Category score of 1 or 2 on day 90 and unfavourable outcome as a Cerebral Performance Category score of 3, 4, or 5 on day 90

^b^In the HYPERION trial used as the source of data for the present study, patients were allocated at random to treatment with hypothermia at 33 °C or normothermia at 37 °C

## Discussion

In the HYPERION trial, which included various population of patients with in and out-of-hospital cardiac arrest and cardiac or non-cardiac causes of arrest, in patients with non-refractory PRS at ICU admission after non-shockable cardiac arrest, on day 90, neither the functional outcome nor survival differed significantly between the groups managed with hypothermia at 33 °C vs. targeted normothermia (37 °C). Functional outcome at day 90 and mortality at day 90 did not differ significantly according to presence or absence of mild-to-moderate PRS. Factors associated with PRS were longer low-flow time, epinephrine treatment during resuscitation, and higher lactate level at ICU admission.

### Effect of temperature on outcome in the PRS population

In parallel of our findings, a post hoc analysis of “TTM1 trial” data demonstrated non-significantly higher all-cause mortality in patients with vs. without PRS at ICU admission [14]. In the HYPERION trial, only about 4 to 20% of deaths were ascribed to PRS, compared to 35% in an earlier study [3], where PRS was also not associated with a higher risk of death from neurological causes in multivariable analysis. A recent larger analysis indicates the opposite with significant association between PRS presence and worse outcome at follow-up [22]. Finally, in a post-hoc analysis of TTM2, During et al. found than hypothermia at 33 °C was not associated with higher incidence of death in patients stratified according to vasopressor support on admission [23].

Opinions conflict about the effects of hypothermia on haemodynamics. In a prospective cohort of 75 patients who required vasopressor therapy, hypothermia at 33–34 °C, whose use was decided by the managing physician, was not associated with worse hemodynamic parameters compared to absence of hypothermia [24]. By contrast, two post hoc analysis of the “TTM1 trial” showed that, compared to 36 °C, 33 °C was associated with a slower heart rate, higher lactate levels, and greater vasopressor requirements, although mortality was not significantly different [13, 14]. In our study, hypothermia was associated with a slower heart rate, but the absence of differences regarding MAP, the SOFA score, and lactate level support the safety of hypothermia in patients with PRS. MAP is a tightly controlled variable, and treatments are given when its values differ from the predefined target. In the HYPERION trial, hemodynamic instability was the main reason for early rewarming in 30% of patients requiring this intervention. Possible associations of hemodynamic fluctuations and rewarming require further evaluation [25].

### Effect of PRS on outcome

The optimal targets and therapeutic interventions in patients admitted to the ICU after cardiac arrest are unclear. In the HYPERION trial, an MAP of 65 mmHg and ScvO_2_ ≥ 70% were considered as reasonable targets, in accordance with guidelines available at the time [19]. Norepinephrine infusion is the cornerstone treatment for achieving the target MAP. However, in a post hoc analysis of the TTH48 trial comparing 33 °C for 24 vs. 48 h, vasopressor requirements were greater in the 48-h group but were not associated with mortality by multivariable analysis [26]. A post hoc analysis of data from a prospective observational study obtained similar findings [27]. More recently, an association was reported linking higher MAP to better outcomes after cardiac arrest [28, 29]. After cardiac arrest, cerebral-blood-flow autoregulation is often impaired or the lower limit right-shifted [30], so that an inadequate MAP target may increase the risk of cerebral hypoperfusion. Two randomized controlled trials found that a higher MAP target failed to improve survival [31, 32]. A recent post hoc analysis of one of these trials showed that the high MAP group had lower concentrations of neurofilament light chains [33], a marker of neurologic prognosis [34] indicating possible benefits of targeting higher MAP with adequate treatments.

### Limitations

One limitation of our study is that the findings apply only to patients with mild-to-moderate PRS, since major hemodynamic instability defined as a continuous epinephrine or norepinephrine infusion > 1 μg/kg/min was an exclusion criterion: only patients with mild-to-moderate shock were included in our analysis [35]. Surprisingly, the global prognosis for patients without PRS (9.1%) or with moderate PRS (7.1%) was poorer than for patients with severe PRS (defined as receiving norepinephrine > 1 µg/kg/min, 13.3%)). This finding could be related to the retrospective design of the study (classification bias). We were unable to perform a pooled analysis, as the centres and definition of PRS differed across studies. The lack of a universal definition of PRS and its severity make comparisons of studies difficult. For example, we found recently than resolution of circulatory failure (defined by the SOFA score at randomization) by day 7 was associated with its severity and the intervention arm [36]. In addition, several origins for shock state can coexist (septic shock related to inhalation pneumonia, cardiogenic shock related to acute coronary occlusion, PRS itself) and complicate interpretation of physiopathology. The prognosis of patients with severe PRS is difficult to assess at ICU admission [35]. Patients were not randomized according to the presence or absence of PRS. The hemodynamic management was not specified in the trial protocol, and physicians applied the usual protocol in their ICU. In addition, these protocols were similarly applied in both groups and the HYPERION trial was stratified according to centres. We did not have details on the hemodynamic management and monitoring in the included patients. Finally, our study may have lacked sufficient power to detect some important associations and furthers analysis of recent “TTM2 trial” [37] will help in delivering care to patients with PRS.

## Conclusions

After non-shockable cardiac arrest, PRS at ICU admission was not associated with day-90 functional outcome or survival. In the sub-group of patients with PRS, outcomes did not differ significantly according to whether hypothermia at 33 °C or normothermia was used.

## Supplementary Information

**Additional file 1:** **Fig. S1.** Study flowchart. **Table S1.** Baseline characteristics and mortality in the groups with vs. without mild-to-moderate postresuscitation shock (PRS) at intensive-care-unit (ICU) admission. **Table S2. **Multivariate logistic regression modelling to identify admission variables associated with a favourable outcome on day 90 in the overall population (*n* = 532).

## Data Availability

The data analysed in this study is subject to the following licenses/restrictions: data sharing on request to corresponding author after Ethics Committee approval. Requests to access these data sets should be directed to Jean-Baptiste Lascarrou, jeanbaptiste.lascarrou@chu-nantes.
