# Supplementary material for: Outcomes of mild-to-moderate postresuscitation shock after non-shockable cardiac arrest and association with temperature management: a post hoc analysis of HYPERION trial data
Source: Ann Intensive Care. 2022 Oct 17;12:96. doi: 10.1186/s13613-022-01071-z (PMC9576832; doi:10.1186/s13613-022-01071-z)
Supplement: Supplementary file 1 — Additional file 1: Fig. S1. Study flowchart. Table S1. Baseline characteristics and mortality in the groups with vs. without mild-to-moderate postresuscitation shock (PRS) at intensive-care-unit (ICU) admission. Table S2. Multivariate logistic regression modelling to identify admission variables associated with a favourable outcome on day 90 in the overall population (n = 532). [file 13613_2022_1071_MOESM1_ESM.doc]

**Figure S1. Study flowchart**

**
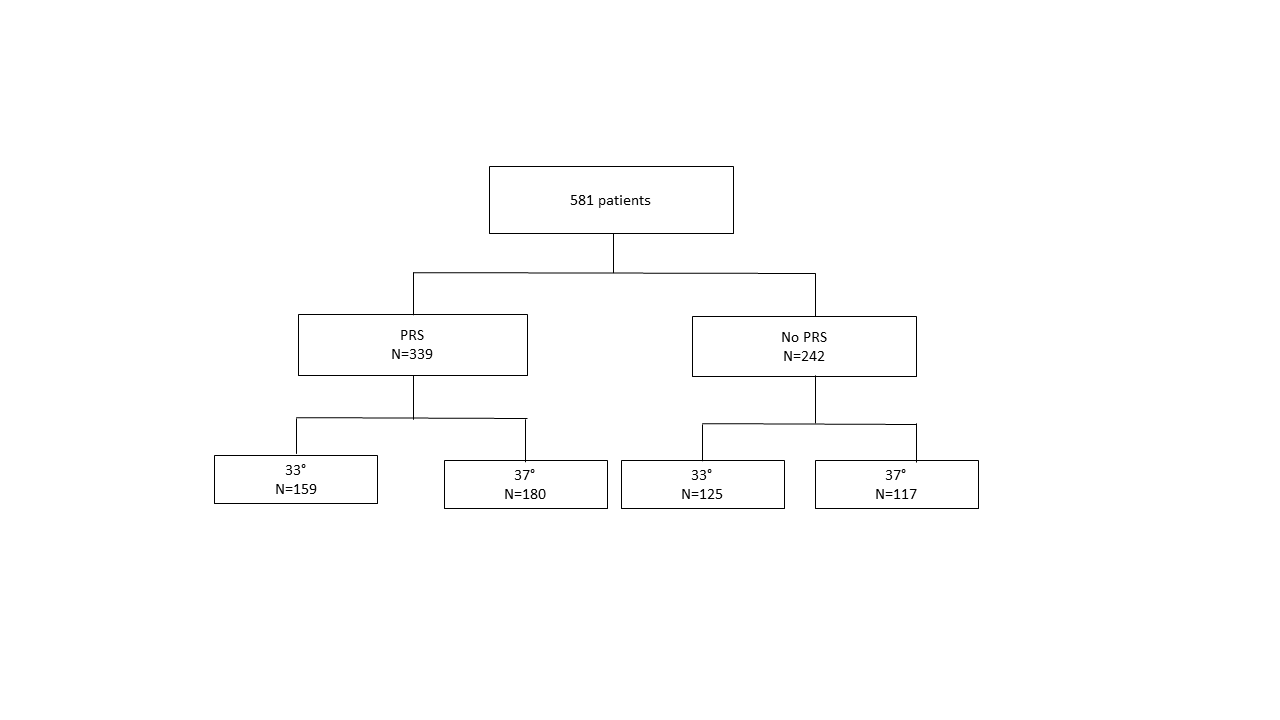
**

**PRS: postresuscitation shock**

**Table S1. Baseline characteristics and mortality in the groups with vs. without mild-to-moderate postresuscitation shock (PRS) at intensive-care-unit (ICU) admission**

| **Characteristics** | **PRS (N=339)** | **No PRS (N=242)** | ***p* value** |
| --- | --- | --- | --- |
| Age, years, median [IQR] | 67.5 [57.8–76.7] | 66.5 [56.5–75.9] | 0.45 |
| Male, n (%) | 210 (62.0) | 163 (67.4) | 0.18 |
| Charlson score, median [IQR] | 4 [2–6] | 4 [2–6] | 0.48 |
| Comorbidities, n (%)  Cardiovascular  Pulmonary | 210 (81.7)  122 (47.5) | 132 (74.6)  82 (46.3) | 0.07  0.81 |
| Cardiac arrest location, n (%)  Home  Public place  Hospital | 169 (49.8)  81 (23.9)  89 (26.3) | 126 (52.1)  46 (19.0)  70 (28.9) | 0.36 |
| Arrest witnessed, n (%) | 324 (95.9) | 223 (92.1) | 0.06 |
| Basic life-support provided by bystander, n (%) | 253 (78.1) | 154 (69.1) | 0.02 |
| First recorded cardiac rhythm (%)  Asystole  Pulseless electrical activity | 273 (87.5)  39 (12.5) | 189 (86.3)  30 (13.7) | 0.69 |
| Cause of cardiac arrest, n (%)  Cardiac  Asphyxia  Anaphylaxis  Neurological  Other medical  Pulmonary embolism  Trauma  Drug poisoning  Drowning | 93 (27.4)  172 (50.7)  7 (2.1)  9 (2.7)  31 (9.1)  17 (5)  3 (0.9)  4 (1.2)  3 (0.9) | 65 (26.9)  148 (61.2)  2 (0.8)  4 (1.6)  11 (4.6)  5 (2.1)  0 (0)  4 (1.6)  3 (1.2) | * |
| GCS score, median [IQR] | 3 [3–3] | 3 [3–4] | 0.0005 |
| Time to randomisation, min, median [IQR) | 236 [182–280] | 214.0 [151–262] | 0.0002 |
| Temperature, °C, median [IQR] | 35.5 [34.3–36.4) | 35.5 [34.6– 36.5] | 0.56 |
| No-flow time, min, median [IQR] | 1 [0–5] | 2.0 [0–6] | 0.22 |
| Low-flow time, min, median [IQR] | 19 [10–28] | 15 [10–22] | 0.0014 |
| Serum lactate at admission, mmol/L, median [IQR] | 6.7 [3.9–10.6] | 5.2 [2.6–7.8] | <.0001 |
| Epinephrine injection, n (%) | 324 (95.6) | 211 (87.2) | 0.0002 |
| Total epinephrine dose, mg, median [IQR] | 3 [2–5] | 3 [2–5] | 0.071 |
| PCI, n (%) | 26 (7.7%) | 16 (6.6%) | 0.63 |
| CAHP score, median [IQR] | 194.2  [172.3–218.1] | 187.7  [160.4–209.0] | 0.0015 |
| Early-onset pneumonia, n (%) | 41 (25.7) | 101 (23.6) | 0.64 |
| CPC, n (%)  1  2  3  4  5 | 16 (4.7)  8 (2.3)  28 (8.3)  0 (0.0)  287 (84.7) | 11 (4.6)  11 (4.6)  25 (10.3)  1 (0.4)  194 (80.1) | 0.32 |
| Favourable outcome (CPC 1 or 2) on day 90, n (%) | 24 (7.1) | 22 (9.1) | 0.38 |
| Day-90 mortality, n (%) | 287 (84.7) | 191 (78.9) | 0.074 |

* Too many categories for a meaningful statistical comparison

GCS: Glasgow Coma Scale; PCI: percutaneous coronary intervention; CAHP: Cardiac Arrest Hospital Prognosis score; CPC: Cerebral Performance Category determined on day 90

**Table S2. Multivariate logistic regression modelling to identify admission variables associated with a favourable outcome on day 90 in the overall population (n=532)**

A favourable outcome was defined as a Cerebral Performance Category score of 1 or 2 on day 90.

1. With interaction factor

|  | OR (95%CI) | *p* value |
| --- | --- | --- |
| Intervention arm: 33° | 1.99 (0.98–4.06) | 0.06 |
| PRS: Yes | 0.98 (0.48–2.04) | 0.97 |
| CAHP score | 0.97 (0.96–0.98) | <0.001 |
| Group*PRS | 1.00 (0.24–4.10) | 0.99 |

1. Without interaction factor

|  | OR (95%CI) | *p* value |
| --- | --- | --- |
| Intervention arm: 33° | 1.99 (0.98–4.06) | 0.06 |
| PRS: Yes | 0.98 (0.49–1.98) | 0.97 |
| CAHP score | 0.97 (0.96–0.98) | <0.001 |

PRS: postresuscitation shock; CAHP: Cardiac Arrest Hospital Prognosis
